# A modelling methodology to assess the effect of insect pest control on agro-ecosystems

**DOI:** 10.1038/srep09727

**Published:** 2015-04-23

**Authors:** Nian-Feng Wan, Xiang-Yun Ji, Jie-Xian Jiang, Bo Li

**Affiliations:** 1 Eco-environment Protection Research Institute, Shanghai Academy of Agricultural Sciences, Shanghai Key Laboratory of Protected Horticultural Technology, Shanghai 201403, China; 2 Ministry of Education Key Laboratory for Biodiversity Science and Ecological Engineering, Institute of Biodiversity Science, Fudan University, Shanghai 200438, China

## Abstract

The extensive use of chemical pesticides for pest management in agricultural systems can entail risks to the complex ecosystems consisting of economic, ecological and social subsystems. To analyze the negative and positive effects of external or internal disturbances on complex ecosystems, we proposed an ecological two-sidedness approach which has been applied to the design of pest-controlling strategies for pesticide pollution management. However, catastrophe theory has not been initially applied to this approach. Thus, we used an approach of integrating ecological two-sidedness with a multi-criterion evaluation method of catastrophe theory to analyze the complexity of agro-ecosystems disturbed by the insecticides and screen out the best insect pest-controlling strategy in cabbage production. The results showed that the order of the values of evaluation index (*R*_CC/CP_) for three strategies in cabbage production was “applying frequency vibration lamps and environment-friendly insecticides 8 times” (0.80) < “applying trap devices and environment-friendly insecticides 9 times” (0.83) < “applying common insecticides 14 times” (1.08). The treatment “applying frequency vibration lamps and environment-friendly insecticides 8 times” was considered as the best insect pest-controlling strategy in cabbage production in Shanghai, China.

Chemical insecticides are often used to repel insect pests in agro-ecosystems. However, the extensive use of chemical insecticides has resulted in many environmental problems, such as ground water contamination, loss of non-target species, reduction of beneficial species, insect pest resistance to insecticides, pest rebound and secondary pest outbreaks and residues in food. In order to protect the environment and ensure food safety, researchers have explored the alternatives to chemical control, and found that biological control, physical control and cultural control are the potential ways of reducing chemical pesticides in agro-ecosystems^1^,^2^,^3^,^4^,^5^. For example, many countries like Indonesia^6^, Norway^7^, Netherlands^8^, Sweden^8^, France^8^, Spain^8^ and Belgium^9^ have already achieved a 50% reduction in the use of chemical insecticides.

Cabbage (*Brassica oleracea*) is regarded as the most important member of the Cruciferae or mustard family, and has been one of the major vegetable crops of the world. The world's first-largest producer of cabbage is China where the annual cultivated area and yield is approximately 100.0 × 10^4^ ha and 10.5 × 10^10^ kg, respectively. In practice, we have found that *Spodoptera exigua* (Hübner), *Spodoptera litura* (Fabricius), *Plutella xylostella* (Linnaeus), *Pieris rapae* (Linnaeus) and aphids are the most serious insect pests. To control these pests, farmers become accustomed to applying chemical insecticides. Official records indicate that chemical insecticide application in cabbage production in Shanghai of China was more than 7.0 kg·ha^−1^ per year (far higher than the national average), which aroused wide attention of the public.

In order to avoid insecticide contamination and ensure food safety, potential insecticide-reducing strategies have been realized in cabbage production at the source in Shanghai suburbs, such as the installation of insect-killing lamps and trap devices (sex attractant and color plastic strips), and the rotation of the use of different classes of environment-friendly insecticides. However, little is known about which of the insecticide-reducing strategies is the optimal or how to identify the best pest-controlling strategy. Therefore, how to evaluate and determine which technique is the best is a challenging task.

To understand the complexity of the agro-ecosystems, Wan et al.^10^ proposed ecological two-sidedness concept to analyze the positive and negative effects of any disturbances on complex ecosystems consisting of economic, ecological and social subsystems^11^,^12^ and developed the *R*_CC/CP_ index borrowed from other existing methodologies to evaluate the superiority of different technologies for pest control^13^,^14^,^15^,^16^,^17^,^18^. It seems that catastrophe theory can be potentially used to evaluate the complex ecosystems disturbed by pest-controlling strategies.

Catastrophe theory is a special topic within the broader domain of nonlinear dynamics systems that was developed by Rene Thom^19^ to study phenomena with non-mechanical discontinuity. Insect pest management can be considered as a process by which sudden and discontinuous changes of state in each of different types of phenomenon in disturbed agro-ecosystems are generated, such as death of insects, resource consumption, pesticide residues and cost input. Thus, catastrophe theory might be a feasible way to shed light on the effects of different pest-controlling strategies on the complex agro-ecosystems. Therefore, this paper used ecological two-sidedness concept based on catastrophe theory to assess the ecological engineering of insect pest-controlling strategies.

## Results

### Values of evaluation indicators

The aim of this study was to screen out the best pest-controlling strategy for cabbage production in Shanghai suburbs. “Negative effect” caused by the candidate strategies was measured by comprehensive negative effect indicators and “positive effect” was measured by comprehensive positive effect indicators (Table 1). According to the reference materials, reference standard values (*x*_0_) of each factor are listed in Tables 2 and 3; and in accordance with the experimental results, the actual values of the indicators (*x_i_*) of comprehensive cost and comprehensive profit for three strategies are also presented in Tables 2 and 3.

### Computational results

In light of ecological two-sidedness concept and catastrophe theory, comprehensive cost (cc) (composed of economic, ecological and social costs) and comprehensive profit (cp) (composed of economic, ecological and social profits) were considered as state variables separately; and different indicators as different control variables. The objective index *u*_cc_ of comprehensive cost or *u_cp_* of comprehensive profit (*A* layer), which is called state variable in catastrophe theory, is the classification of pest-controlling strategies. There are three indicators in the second evaluation level (*B* layer), including economic cost (*u*) or economic profit (*u′*), ecological cost (*v*) or ecological profit (*v′*), and social cost (*w*) or social profit (*w′*). In the root evaluation level (*C* layer), nine indicators for comprehensive cost and ten indicators for comprehensive profit were involved.

The initial data (*x_i_*) from the *C* layer were transformed into dimensionless data with value from 0 to 1. To get dimensionless data (Tables 2 and 3), each of initial data was divided by each reference standard data (*x*_0_). There were two control variables for social cost and social profit which were both adopted by cusp catastrophe model, three control variables for ecological cost which were adopted by swallowtail catastrophe, and four control variables for economic cost, economic profit and ecological profit which were all adopted by butterfly catastrophe model. The evaluation results of the different strategies in *C* layer are presented in Table 4 with the analysis of catastrophe models. According to the complementary principle, the evaluation results of the different strategies in *B* layer and *A* layer were obtained and are given in Tables 5 and 6. Hence, *W_cc/cp_*, matrix of ratio of comprehensive cost to comprehensive profit (*R_cc/cp_*) for three treatments was obtained by *W_cc/cp_* = (*R_cc/cp_*,_ 1_, *R_cc/cp_*,_ 2_, *R_cc/cp_*,_ 3_)^T^ = (0.75/0.94, 0.77/0.93, 0.85/0.79)^T^ = (0.80, 0.83, 1.08)^T^ (Table 6). According to ecological two-sidedness concept, the lower the value of *R_cc/cp_* was, the more reasonable the candidate strategy was^10^,^15^. Therefore, the order of superiority of pest-controlling strategies was treatment one > treatment two > treatment three, i.e., the treatment “applying frequency vibration lamps and environment-friendly insecticides 8 times” should be identified as the best insect pest-controlling strategy in cabbage production in Shanghai, China.

### Experimental results

The increasing order of labor cost was treatment one (applying frequency vibration lamps and environment-friendly insecticides 8 times) < treatment two (applying trap devices and environment-friendly insecticides 9 times) < treatment three (control areas, no lamps, no trap devices, applying common insecticides 14 times). The orders of insecticide cost, instrument depreciation cost, active insecticide component dosage, and insecticide stress on society were all the same. The insecticide residues in cabbages were significantly greater in the control than other treatments but not significantly different among treatments, and the same order was presented in the indicator of the efficacy of killing natural enemies. Cost of non-chemical control was just inputted in treatment one and treatment two; three treatments were given the same irrigation water consumption (Table 2).

The order of income from cabbage production was treatment one > treatment two > treatment three; the order of insecticide safety was treatment one = treatment two > treatment three, and the same orders were for time-saving, increased value per unit investment of cabbage commodity, production value created by every labor force and percentage of farmer acceptance and life cycle of technology. The ratio of the abundance of beneficial arthropods to the one of harmful arthropods was significantly higher both in treatment one and treatment two than the control (Table 3), and the same orders were presented in the ratio of the abundance of neutral arthropods to the one of harmful arthropods and the arthropod diversity.

## Discussion

Chemical insecticides might be the most effective, convenient and reliable method for pest suppression once the pest situation is out-of-hand. However, chemical insecticides often cause serious environmental and food safety problems, so it might be best to minimize the use of chemical insecticides, or use them carefully or selectively^3^,^4^,^5^. In order to control insect pests and to avoid the soil contamination by chemical insecticides, researchers have explored the strategies such as biological control^20^, physical control^21^ and cultural control^22^,^23^,^24^. Here we used the strategies of optimizing insecticide variety structure (the use of eight slightly toxic insecticides was rotated, and the four moderately toxic insecticides, 48% Chlorpyrifos EC, 20% fenvalerate EC, 18% Bisultap AS and 2.5% Deltamethrin, were excluded), installing trap devices and installing lamps to control insect pests in cabbage fields.

Different strategies use varying doses of insecticides, and might affect the richness and abundance of arthropods. Our study indicated that arthropod communities were more diverse and stable, and species richness and abundance of beneficial arthropods were significantly higher in the two treatments with lamps or trap devices than in control. Compared to those in the control, in the strategy of “lamps + environment-friendly insecticides 8 times” and the strategy of “trap devices + environment-friendly insecticides 9 times”, the arthropod diversity increased respectively by 19.3% and 21.9%, the beneficial: harmful arthropod ratio increased by 77.5% and 80.4%, the beneficial: harmful arthropod ratio increased by 56.7% and 67.2%, while the active ingredients of insecticides decreased respectively by 52.0% and 51.6% in the two strategies.

One of the most important sections of catastrophe theory is that catastrophe progression method has no need to assign the weight to each indicator and has a simple calculation procedure, which has been widely applied to the comprehensive evaluation^25^,^26^,^27^. Here we used this method to assess the ecological engineering of insect pest-controlling strategies in agro-ecosystems, which can help to solve the problem of pesticide pollution. The information provided here is useful for the application of catastrophe theory to the field of pest management assessment. However, further attention should be paid to the establishment of the fuzzy membership functions of underlying indicators, the dissociation of the indicator system and the priority of indicators from each level.

Ecological two-sidedness approach proposed by Wan et al.^10^ has been applied to the ecological assessment of vegetable ecosystems^15^, paddy ecosystems^13^,^17^ and protected horticultural fields^14^,^16^, as well as to the assessment of the combined reduction of chemical pesticides and chemical fertilizers for low-carbon agriculture^18^. Here we adopted this theory to analyze the effects of different pest-controlling strategies on cabbage-producing ecosystems, and established a comprehensive evaluation system based on catastrophe theory, but it might also have the potential to apply other theories to treat such problems.

The methodology developed here reduces the subjectivity of decision-makers, farmers and experts. The maximum value of the *R_cc/cp_* was 1.08 in the control, implying that the control was the worst strategy for pest control in cabbage production in Shanghai. Our conclusion here was similar to that of the expert group from local practices. However, it is still necessary to further determine if a more scientific and reasonable evaluation system has been identified, and to validate that system in practice. Also, we need to further improve both the strategies and the assessment methods to optimize the identification of pest-controlling strategies for the cabbage production.

## Methods

### Experimented materials

Cabbage variety (Meimao II), produced by Xiehe company of Japan; frequency vibration insecticidal lamps (PS-15 II type), manufactured by Jiaduo company limited of Henan province of China; manual pesticide sprayers (3WBS-16 type), manufactured by Qiangye sprayer factory of Luozhuang district of Linyi city of Shandong province of China; trap devices (tobacco cutworm sex pheromone, beet armyworm sex pheromone, diamond back moth sex pheromone, plastic tubes for suspending sex pheromone, yellow and blue plastic strips), manufactured by Zhangzhou Enjoy Technology Company Limited of China. Insecticides included 10% Imidacloprid WP, 10% Chlorfenapyr SC, 20% Tebufenozide SC, 15% Indoxacarb SC, 40% Phoxim EC, 25% Buprofezin WP, 48% Chlorpyrifos EC, 2.5% Deltamethrin EC, 4.5% Beta cypermethrin EC, 18% Bisultap AS, 20% Fenvalerate EC and ACNPV SC.

### Experiment design and method

Experiments were conducted by adopting a series of individual 1 ha plots in cabbage fields in Modern Agriculture Park of Songjiang District, Shanghai of China (121.13°E, 30.94°N) from April to October in 2006. Three treatments were included in the experiment design. In treatment one, 1.0-m high frequency vibration lamps were installed 30 days after the transplanting of cabbage. In treatment two, lamps were not adopted but trap devices including sex pheromone lures and color (yellow and blue) trap strips were used throughout the entire growing period of cabbages after transplanting. However, lamps or trap devices were not installed in the control (treatment three) in which conventional insect pest-controlling strategies were used by farmers with empirical planting. Each treatment and the control were replicated three times.

Slightly toxic insecticides (10% Imidacloprid WP, 10% Chlorfenapyr SC, 20% Tebufenozide SC, 15% Indoxacarb SC, 40% Phoxim EC, 25% Buprofezin WP, 4.5% Beta cypermethrin EC and ACNPV SC) were used in treatments one and two, while the moderately toxic insecticides (48% Chlorpyrifos EC, 2.5% Deltamethrin EC, 20% Fenvalerate EC and 18% Bisultap AS) and slightly toxic ones (10% Imidacloprid WP and 40% Phoxim EC) were done in the control. In treatment and control fields, insecticides were appropriately used in accordance with the population dynamics of insect pests.

The total growing period of cabbages was approximately 100 days, seeds sown in early July, seedlings transplanted at a density of 45000 plants·ha^−1^ in early August, cabbages harvested in middle October. The sex pheromone was placed inside a capillary vessel (0.1-m long and 1.5-mm caliber). One each tobacco cutworm, beet armyworm and diamondback moth sex pheromone trap was installed at 20 m intervals in each direction in each plot of treatment two, was hung 1.0 m above the ground level, and was replaced once per month. Seventy-five yellow plastic strips per hectare and seventy-five blue plastic strips per hectare were installed with equidistance in each plot of treatment two, and were suspended 0.2 m above the plants. Color strips' dimensions were all 0.3-m long, 0.4-m wide, 1.5-mm thick, with sticky jelly well distributed on. Mini pests like aphids and whiteflies tend towards yellow things while thrips tend towards blue things so that they stick to death on the plastic strips, and the aim of pest control can be achieved.

All the treatments were given similar management of water and fertilizers during the whole period of cabbage growth. Weeds were managed by hand pulling. Arthropods were systematically scouted and sampled at about 20–30 d intervals in all fields. “Z” style sampling with 5 dots of 100 plants was employed at random from seedling stage to harvesting stage^16^. To ensure the continuity and reliability of the statistics, we routinely recorded the service condition of the agricultural resources in each of the plots, the planting and growing management procedures, cost accounting factors, the sales status of cabbages, etc. All data presented were analyzed with Microsoft Excel and SPSS16.0 softwares.

### Determination of evaluation indicators

**Figure 1:**
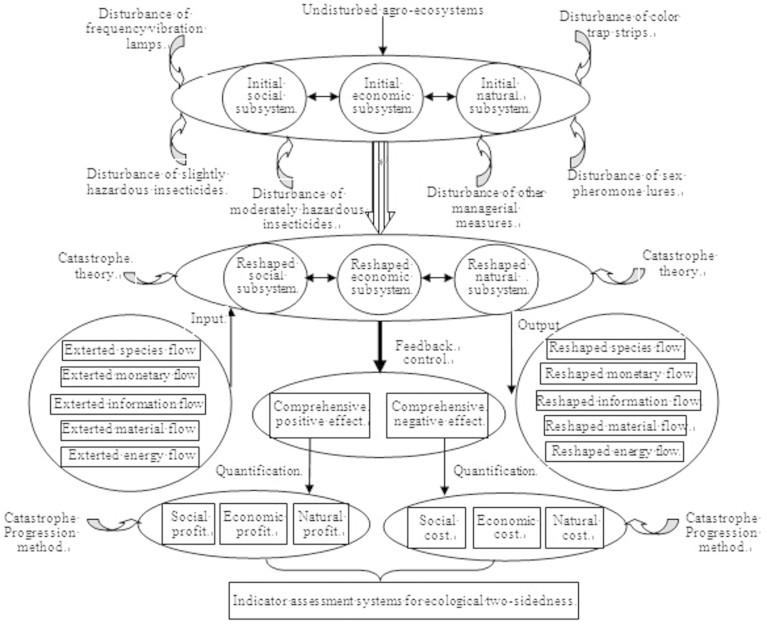
The framework of understanding the agro-ecosystems disturbed by the insect pest-controlling strategies.

According to the idea of ecological two-sidedness approach, the agro-ecosystems disturbed by the insect pest-controlling strategies either acquire profit or incur a cost (Fig. 1). The complex ecosystem is composed of three subsystems (economic, ecological and social subsystems) with mutual restrictions. The relevant effect indicators should be considered when measuring the standard in the complex ecosystem. These indicators need to address the following questions: whether the economic subsystem is beneficial, whether the ecological subsystem is stable, and whether the social subsystem is rational^17^. According to above goals, a specific indicator system should be confirmed that maximizes the comprehensive profit and minimizes the comprehensive cost. As the complex ecosystem in cabbage fields is measured by indicators of the three subsystems, constructing an indicator system for pest control should abide by the following criteria: (1) indicators should reflect economic, ecological and social aspects, economic cost and profit, resources and time consumption, acceptance degree, as well as the impact of candidate strategies on society and nature^17^; (2) indicators should reflect the short- and long-term profits, partial and whole cost that candidate strategies can bring^15^; (3) quantitative indicators should be adopted as far as possible, and subjective deviation should be avoided^17^; (4) comprehensive indicators should be used and each indicator should keep independent without directly interactive relationships^16^,^28^; (5) the indicators should complement each other, and the structure produced by the indicators should reflect fully and even completely its various functional characteristics and be constituted completely^29^; (6) indicators should reflect the diversity, stability and structure characteristics of arthropods^14^. According to the above criteria, the evaluation indicators for insect pest-controlling strategies in cabbage fields were summarized and listed in Table 1. Indicator standards refer to safe and health standards of agricultural produce issued by Shanghai Standardization Association.

Different strategies might lead to different economic costs which include the labor cost, insecticide cost, instrument depreciation cost, cost of non-chemical control (lamp and electric cost, trap device cost), etc. Positive economic effect indicators were split into either direct or indirect. Direct effects included cabbage income, cabbage commodity increase value per unit investment and production value created by the labor force, while indirect effects included saving time (the lowest person workdays per hectare was 390.0 in cabbage production).

Negative ecological effect indicators reflected the adverse impact of strategies on the ecological subsystem. The effect of each strategy on natural enemies was measured by the difference between 3.3 and the actual natural enemy diversity in all treatments and control fields. The greater the percentage of difference between the two diversity values, the stronger the effect of the strategy for damaging natural enemies^15^. Enzyme inhibition rate method was adopted to detect pesticide residues in cabbage produce. The active insecticide component dosage played an important role in the preservation of natural environment and the maintenance of food safety.

Positive ecological indicators reflected the profitable effect of strategies on the ecological subsystem. Beneficial and harmful arthropods are the base of the food web in the tritrophic interactions in agro-ecosystems, both of which play important roles in the regulation of harmful arthropods; arthropod diversity maintained the diversity and stability of agro-ecosystems; insecticide safety was measured by the geometric averages of the LD_50_ for acute oral toxicity of insecticides on rats.

Social effect indicators reflected the impact of strategies on the social subsystem. A questionnaire survey was conducted to obtain information regarding farmers' acceptance of various strategies. Life cycle of technology had a profound effect on agricultural production which involved some other sections of social production. The indicator of insecticide stress on society was measured according to the total insecticide dosage, as the more insecticides were used, the greater stress of the insecticides on society might be. Irrigation water consumption was measured in reference to actual volume of water consumed.

### Evaluation theory and methods

Catastrophe theory, a new mathematical discipline, is a special branch of dynamical systems theory and can be adopted to evaluate the status and analyze the development trends, known as “the mathematical revolution after calculus”^30^. Catastrophe theory has been widely used, and one common application is the use of the catastrophe progression method derived from catastrophe theory to solve multiple criteria decision making problems^27^. In the multi-criteria evaluation method based on catastrophe theory, the dependency of state variables on control variables is determined by the catastrophe fuzzy membership functions, rather than weights assigned by the users, and different control variables have different impacts on state variables^25^.

The general evaluation process of catastrophe progression method consists of the following steps. First, the complex ecosystem is divided into three subsystems (economic, ecological and social subsystems) with different evaluation indicators in accordance with the inner mechanisms of the complex ecosystem. Next, the initial data from the underlying layers are normalized by the integration of catastrophe theory and fuzzy mathematics, and to realize this, multidimensional catastrophe fuzzy membership functions should assign values ranging from 0 to 1 to resolve the incomparability of various initial data generated by differences in the data span and dimensions. Finally, the total catastrophe fuzzy membership functions of the complex ecosystems are obtained by the normalized data^25^.

Thom has presented seven fundamental types of catastrophe models, among which the models of elliptic umbilic catastrophe, hyperbolic umbilic catastrophe and parabolic umbilic catastrophe are under the behavior variation of two dimensions and the models of fold catastrophe, cusp catastrophe, swallowtail catastrophe and butterfly catastrophe are commonly applied. The four potential functions and normalization formulas are as follows:
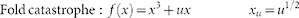

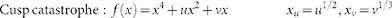

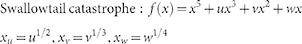

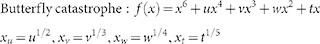
where *f* (*x*) is the potential function of the state variable *x*, and *u*, *v*, *w* and *t* are control variables of the state variable.

The catastrophe progression of each control variable can be computed from the initial fuzzy subordinate function, based on normalization formulas. When performing recursive computations, non-complementary principle (the principle of minimum values) or complementary principle (the principle of mean values) is selected after the determination of whether or not the indicators are complementary to each other or interchangeable within one subsystem^25^. The non-complementary principle means that the control variables of a system, such as *u*, *v*, *w*, and *t* cannot offset each other. Therefore, when finding the value of the state variable *x* using the normalization formulas, the smallest of the state variable values corresponding to the control variables (*x_u_*, *x_v_*, *x_w_* and *x_t_*) is selected as the state variable value of the whole system. The complementary principle means that the control variables complement each other so that each of them tends to reach the average value, i.e., *x* = (*x_u_* + *x_v_* + *x_w_* + *x_t_*)/4. Hierarchically doing the calculation in the same way, the value of the overall catastrophe subordinate function can be obtained^27^.

## Author Contributions

W.-N.F. and J.-J.X. conceived and designed the experiment; W.-N.F., J.-X.Y. and J.-J.X. performed the experiment; W.-N.F. and L.B. analyzed the data; W.-N.F., J.-J.X. and L.B. wrote the paper.

## Figures and Tables

**Table 1:** Comprehensive cost and comprehensive profit indicators for insect pest-controlling strategies in cabbage production

Sub-object layer (B layer)	Indicator layer (C layer)	Sub-object layer (B layer)	Indicator layer (C layer)
Economic cost (*u*)	Labor cost (*u*_1_)	Economic profit (*u′*)	Income from cabbage production (*u′*_1_)
	Insecticide cost (*v*_1_)		Time-saving(*v′*_1_)
	Cost of non-chemical control (*w*_1_)		Increased value per unit investment of cabbage commodity (*w′*_1_)
	Instrument depreciation cost (*t*_1_)		Production value created by every labor force (*t′*_1_)
Ecological cost (*v*)	Killing natural enemy (*u*_2_)	Ecological profit (*v′*)	Beneficial: harmful arthropod ratio (*u′*_2_)
	Insecticide residue in cabbages (*v*_2_)		Neutral: harmful arthropod ratio (*v′*_2_)
	Active insecticide component dosage (*w*_2_)		Arthropod diversity index (*w′*_2_)
	—		Insecticide safety (*t′*_2_)
Social cost (*w*)	Insecticide stress on society (*u*_3_)	Social profit (*w′*)	Percentage of farmer acceptance (*u′*_3_)
	Irrigation water consumption (*v*_3_)		Life cycle of technology (*v′*_3_)

Note: “–” means no indicator.

**Table 2:** Indicator data of comprehensive negative effect for insect pest-controlling strategies in cabbage production

Comprehensive cost	Unit	Indicator description	Reference standard value	Treatment one		Treatment two		Treatment three	
			*x*_0_	*x_i_*	*x_i_*/*x*_0_	*x_i_*	*x_i_*/*x*_0_	*x_i_*	*x_i_*/*x*_0_
Economic cost	RMB·ha^−1^	Labor cost	7800.00	5400.00	0.69	5700.00	0.73	6600.00	0.85
	RMB·ha^−1^	Insecticide cost	1200.00	661.80	0.55	667.80	0.56	720.00	0.60
	RMB·ha^−1^	Cost of non-chemical control	1000.00	48.00	0.05	364.95	0.36	0.00	0.00
	RMB·ha^−1^·a^−1^	Instrument depreciation cost	190.00	96.00	0.51	108.00	0.57	168.00	0.88
Ecological cost	%	Killing natural enemy	50.00	10.30 ± 1.43b	0.21	8.48 ± 0.97b	0.17	45.35 ± 1.51a	0.91
	%	Insecticide residue in cabbages	10.00	4.27 ± 0.12b	0.43	4.57 ± 0.28b	0.46	9.97 ± 0.35a	1.00
	kg·hm^−2^	Active insecticide component dosage	2.00	0.70	0.35	0.71	0.36	1.47	0.74
Social cost	kg·ha^−1^	Insecticide stress on society	10.00	5.25	0.53	5.40	0.54	7.20	0.72
	m^3^·ha^−1^	Irrigation water consumption	9000.00	7500.00	0.83	7500.00	0.83	7500.00	0.83

**Table 3:** Indicator data of comprehensive positive effect for insect pest-controlling strategies in cabbage production

Comprehensive cost	Unit	Indicator description	Reference standard value	Treatment One		Treatment Two		Treatment Three	
			*x*_0_	*x_i_*	*x_i_*/*x*_0_	*x_i_*	*x_i_*/*x*_0_	*x_i_*	*x_i_*/*x*_0_
Economic profit	RMB·ha^−1^	Cabbage income	80000.00	75274.50 ± 71.30a	0.94	75175.50 ± 73.80a	0.94	74003.50 ± 258.90a	0.93
	workday·ha^−1^	Time-saving	150.00	120.00	0.80	105.00	0.70	60.00	0.40
	—	Increased value per unit investment of cabbage commodity	1.00	0.82	0.82	0.76	0.76	0.61	0.61
	RMB·ha^−1^	Production value created by every labor force	300.00	278.80	0.93	263.80	0.88	224.30	0.75
Ecological profit	—	Beneficial: harmful arthropod ratio	16.00	15.67 ± 0.66a	0.98	15.93 ± 0.49a	1.00	8.83 ± 0.20b	0.55
	—	Neutral: harmful arthropod ratio	16.00	13.43 ± 0.58a	0.84	14.33 ± 0.35a	0.90	8.57 ± 0.29b	0.54
	—	Arthropod diversity index	5.00	4.46 ± 0.04a	0.89	4.56 ± 0.03a	0.91	3.74 ± 0.09b	0.75
	mg·kg^−1^	Insecticide safety	2500.00	2446.20	0.98	2446.20	0.98	541.50	0.22
Social profit	%	Percentage of farmer acceptance	100.00	88.46	0.88	84.62	0.85	80.77	0.81
	a	Life cycle of technology	10.00	6.00	0.60	5.00	0.50	1.00	0.10

Note: “–” means symbol without unit; increased value per unit investment of cabbage commodity = (cabbage income of technology optimization area in unit area – cabbage income of conventional pest-controlling area in unit area) ÷ investment value of technology for s cabbage production in unit area; production value created by every labor force, refers to the economic income of cabbages created by every workday.

**Table 4:** Catastrophe progression values of indicator layer for insect pest-controlling strategies in cabbage production

Sub-object layer (*B* layer)	Indicator layer (*C* layer)	Treatment one	Treatment two	Treatment three
Economic cost (*u*)	Labor cost (*u*_1_)	0.83	0.85	0.92
	Insecticide cost (*v*_1_)	0.82	0.82	0.84
	Cost of non-chemical control (*w*_1_)	0.47	0.77	0.00
	Instrument depreciation cost (*t*_1_)	0.87	0.89	0.97
Ecological cost (*v*)	Killing natural enemy (*u*_2_)	0.46	0.41	0.95
	Insecticide residue in cabbages (*v*_2_)	0.75	0.77	1.00
	Active insecticide component dosage (*w*_2_)	0.77	0.77	0.93
Social cost (*w*)	Insecticide stress on society (*u*_3_)	0.73	0.73	0.85
	Irrigation water consumption (*v*_3_)	0.94	0.94	0.94
Economic profit (*u′*)	Cabbage income (*u′*_1_)	0.97	0.97	0.96
	Saving time (*v′*_1_)	0.93	0.89	0.74
	Increased value per unit investment of cabbage commodity (*w′*_1_)	0.95	0.93	0.88
	Production value created by every labor force (*t′*_1_)	0.99	0.97	0.94
Ecological profit (*v′*)	Beneficial: harmful arthropod ratio (*u′*_2_)	0.99	1.00	0.74
	Neutral: harmful arthropod ratio (*v′*_2_)	0.94	0.97	0.81
	Arthropod diversity index (*w′*_2_)	0.97	0.98	0.94
	Insecticide safety (*t′*_2_)	1.00	1.00	0.74
Social profit (*w′*)	Percentage of farmer acceptance (*u′*_3_)	0.94	0.92	0.90
	Life cycle of technology (*v′*_3_)	0.84	0.79	0.46

**Table 5:** Catastrophe progression values of sub-object layer for insect pest-controlling strategies in cabbage production

Object layer (*A* layer)	Sub-object layer (*B* layer)	Treatment one	Treatment two	Treatment three
Comprehensive cost (*u*_cc_)	Economic cost (*u*)	0.75	0.83	0.68
	Ecological cost (*v*)	0.66	0.65	0.96
	Social cost (*w*)	0.84	0.84	0.90
Comprehensive profit (*u*_cp_)	Economic profit (*u′*)	0.96	0.94	0.88
	Ecological profit (*v′*)	0.98	0.99	0.81
	Social profit (*w′*)	0.89	0.86	0.68

**Table 6:** Catastrophe progression values of object layer for insect pest-controlling strategies in cabbage production

Object layer (*A* layer)	Treatment one	Treatment two	Treatment three
Comprehensive cost (*u*_cc_)	0.75	0.77	0.85
Comprehensive profit (*u*_cp_)	0.94	0.93	0.79
